# Effect of HSV-2 on population-level trends in HIV incidence in Uganda between 1990 and 2007

**DOI:** 10.1111/tmi.12176

**Published:** 2013-09-10

**Authors:** Samuel Biraro, Anatoli Kamali, Richard White, Alex Karabarinde, Juliet Nsiimire Ssendagala, Heiner Grosskurth, Helen A Weiss

**Affiliations:** 1 Medical Research Council/Uganda Virus Research Institute, Uganda Research Unit on AIDSEntebbe, Uganda; 2 London School of Hygiene and Tropical Medicine, Medical Research Council Tropical Epidemiology GroupLondon, UK; 3 Department of Epidemiology and Population Health, London School of Hygiene and Tropical MedicineLondon, UK

**Keywords:** HIV, HSV-2, prevalence, incidence, trends

## Abstract

**Objective** To assess the long-term effects of population-level HSV-2 infection on HIV incidence.

**Methods** Data from a population-based cohort in south-western Uganda were used to estimate HIV incidence from 1990 to 2007. Stored blood samples were tested for HSV-2, and the impact of HSV-2 prevalence and incidence on HIV incidence was estimated by calculating population attributable fractions (PAFs). The association between population-level annual HIV incidence and annual HSV-2 incidence/prevalence was analysed using linear regression.

**Results** HIV incidence declined over time among men, from 8.72/1000 person-years (pyr) in 1990 to 4.85/1000 pyr in 2007 (*P*-trend <0.001). In contrast, there was no decline in HIV incidence among women (4.86/1000 pyr in 1990 to 6.74/1000 pyr in 2007, *P*-trend = 0.18). PAFs of incident HIV attributable to HSV-2 were high (60% in males; 70% in females). There was no evidence of an association between long-term trends in HIV incidence and HSV-2 prevalence or incidence.

**Conclusion** Assuming a causal relationship, a substantial proportion of new HIV infections in this population are attributable to HSV-2. The study did not find an effect of HSV-2 prevalence/incidence on trends in HIV incidence. HIV incidence did not vary much during the study period. This may partly explain the lack of association.

## Introduction

Few African population-based studies have reported trends in HIV incidence over time (Shafer *et al*. 2008). Direct estimates of HIV incidence are useful measures of progress in HIV prevention, as unlike HIV prevalence estimates, they do not depend on treatment and mortality parameters (Mahy *et al*. 2009; Joint United Nations Programme on HIV/AIDS (UNAIDS) & The Global HIV/AIDS Program (GHAP) The World Bank 2009). However, direct determination of incidence is expensive and logistically difficult. In this study, we report results from a long-term population-based cohort in rural south-western Uganda to directly estimate incidence from 1990 to 2007 and to examine the association between HSV-2 infection and risk of HIV acquisition in this population.

Clinical, biological and epidemiological evidence suggests that HSV-2 infection increases the risk of HIV acquisition (Glynn *et al*. 2009) and transmission (Shacker *et al*. 2002; Nagot *et al*. 2007; Zuckerman *et al*. 2007; Baeten *et al*. 2008; Dunne *et al*. 2008; Delany *et al*. 2009) at an individual level, through clinical and subclinical reactivations of HSV-2. Randomised controlled trials of HSV-2 suppressive treatment have not found an effect on HIV incidence rate. However, lack of effect may have been the result of poor adherence to study drug, suboptimal drug concentrations at the given dose, or ineffective study drug (Celum *et al*. 2008; Watson-Jones *et al*. 2008; Zhu *et al*. 2009). To our knowledge, there have been no empirical studies of the association between HIV incidence and HSV-2 infection at population level. Mathematical models have estimated that, in a mature HIV epidemic, HSV-2 infection may account for 25–50% of new HIV infections (Freeman *et al*. 2007; Abu-Raddad *et al*. 2008), that a hypothetical prophylactic HSV-2 vaccine may reduce HIV incidence by 30–40% after 20 years (Freeman *et al*. 2009), and that long-term HSV-2 suppressive therapy may reduce risk of onward transmission of HIV and reduce HIV incidence by 30% (White *et al*. 2008). These studies reflect the potential of effective HSV-2 control on HIV incidence. In this study, we estimate the proportion of new HIV infections attributable to HSV-2 infection and analyse the association between population-level trends in HIV incidence and HSV-2 prevalence and incidence.

## Methods

### Study population

Details of the cohort have been published previously (Shafer *et al*. 2008). Briefly, the study is located in a subcounty in south-western Uganda, in which household surveys of socio-demographic and behavioural characteristics, as well as HIV serostatus of all consenting residents aged 13 years and above have been conducted annually since 1989. The cohort was initially comprised of about 10 000 people residing in 15 neighbouring villages (the old villages) but was expanded at the 1999/2000 survey to approximately 18 000 people in 25 villages. The average annual participation rate is about 60–65%. Community sensitisation activities precede each survey round, including local council briefings and village meetings. All households are visited by, in turn, the mapping, census and survey teams. Consenting residents are interviewed at home in the local language by trained survey staff and provide a blood sample for HIV testing. Each annual survey begins in November and ends in October the following year. This study used data and stored blood samples collected between 1990 and 2007 from participants aged 13–59 years. For simplicity, we denote survey rounds by the year in which the survey ends (e.g. data collected from November 1989 to October 1990 are denoted 1990).

### Laboratory methods

HIV infection at each visit was determined using two parallel ELISA tests (Wellcozyme HIV-1 recombinant VK 56/57, Murex Biotech, Dartford, UK; and Recombigen HIV-1/2, Trinity Biotech, Galway, Ireland) confirmed with Western blot in case of first-time positives or discordant ELISA (Van der Paal *et al*. 2007). HSV-2 was determined using the Kalon ELISA (Kalon Biological, UK) with cut-off optical density for a positive result at 1.5 (Biraro *et al*. 2011). To minimise unnecessary testing, all samples were tested for HSV-2 at key survey rounds (1990, 1994, 2000, 2007). Samples from participants testing positive at these rounds were back-tested in the previous rounds until a negative result was detected.

### Statistical methods

Analyses were restricted to the 15 villages with data from the whole follow-up to allow estimation of changes over time. An a priori decision was taken to stratify analyses by gender because incidence rates and risk factors for HIV infection differ by sex. Annual HIV incidence rates were estimated from 1990 to 2007. Participants were censored at the earliest of the last HIV-negative test or estimated date of seroconversion (estimated as the mid-point of the last negative and first positive result). Both observed and smoothed incidence rates were plotted for easier visual interpretation of trends. Smoothing was carried out using the locally weighted scatterplot smoothing (LOWESS) procedure (Cleveland & Devlin 1988).

HSV-2 test results were available from 48.9% of the eligible resident population overall, and from 48.2%, 41.9%, 50.6% and 53.9% in 1990, 1994, 2000 and 2007, respectively. To minimise bias due to missing data, HSV-2 prevalence was estimated using multiple imputation (Sterne *et al*. 2009). Data were assumed to be ‘missing at random’ as HSV-2 data were missing mainly because participants chose not to provide a blood sample due to study fatigue, due to absence from home, or lack of availability of samples (Wagner *et al*. 1994; Kamali *et al*. 1999). Variables included in the multiple imputation were sex, age, marital status, HIV status and observed HSV-2 status results. These were selected because they were the main independent predictor variables for HSV-2 infection that were available in the data set. Annual HSV-2 incidence rates were calculated using an approach similar to that for HIV incidence described above, on the basis of actual HSV-2 test result, rather than longitudinal or multiple imputation. However, unduly long intervals between the last negative and first positive results will result in imprecise estimated dates of seroconversion and may cause an artificially high estimate of incidence in the periods corresponding to the mid-points. To examine this potential bias, sensitivity analyses were conducted by comparing incidence estimates from analyses including all participants irrespective of the length of the interval between last negative and first positive test with estimates from analyses limited to participants with intervals less than 4, 3 and 2 years, respectively. Estimates for annual HSV-2 incidence were similar, so all participants with interviews of up to 4 years retained in the analysis.

To study the effect of HSV-2 infection on HIV incidence, crude and adjusted incidence rate ratios (IRR) and 95%CI were estimated using Poisson regression. A hierarchical modelling approach was used (Victora *et al*. 1997). An a priori decision was made to adjust all analyses for time period and age group, as risk of HIV incidence is likely to be associated with these factors. Four time periods were defined. These were two 5-year periods in the 1990s when HIV prevalence/incidence was known to have declined in this population and two periods in the 2000s when declines in HIV prevalence/incidence were no longer observed (Shafer *et al*. 2008). The time periods are 1990–1994, 1995–1999, 2000–2004 and 2005–2007. *P*-values for association were estimated using the likelihood ratio test. As the primary exposure of interest, HSV-2 was retained in the model irrespective of the level of association with HIV incidence. Initially, the association between HIV and socio-demographic factors was analysed, and factors with *P* < 0.15 were fitted in a core socio-demographic model and retained if they remained independently associated with HIV (*P* < 0.15). Subsequently, each behavioural factor was adjusted for this core socio-demographic model. Behavioural factors in the model were then removed one at a time and retained if significant at *P* < 0.15. Finally, as the primary exposure of interest, HSV-2 was added into the model and other factors retained if independently associated with HIV (*P* < 0.15). Factors that remained with *P* < 0.05 in the final model were considered to be independently associated with incident HIV infection. Demographic factors included in the analysis were period in time, age, level of education, marital status and religion; whereas behavioural factors were age at first sex, number of lifetime sexual partners, number of sexual partners in the last year. As risky behaviour is known to increase risk of HIV incidence, risk factor analysis for behavioural variables was limited to sexually active participants to compare risk for different levels of risky behaviour, but all participants were included in the final model.

To estimate the impact of HSV-2 on HIV incidence at the population level, population attributable fractions (PAFs) were estimated using adjusted incidence rate ratios (aIRR) for HSV-2 from Poisson regression models for HIV incidence adjusting for the effect of other factors that were found to be independently associated with HIV incidence at the individual level (Brady 1998). These factors included increasing age, being an internal migrant, not being Muslim, being presently or previously married, and being HSV-2 infected, as well as time period (men only) and belonging to tribe other than Muganda (women only). PAFs were estimated separately by sex and age group. The age group cut-off was defined as the median age at HIV seroconversion to maximise statistical power. The median age at HIV seroconversion was found to be 28.7 years overall and 31 in men and 26 in women. As both HIV and HSV-2 are sexually transmitted infections and may be acquired simultaneously, PAFs were estimated for incident HSV-2 (i.e. seroconversion to HSV-2 in the same time period as HIV seroconversion) and prevalent HSV-2 (HSV-2 infection prior to HIV seroconversion), separately.

Analysis for risk of acquisition examined the effect of HSV-2 prevalence/incidence on HIV incidence in the same gender, whereas analysis for risk of transmission examined effect HSV-2 prevalence/incidence from one gender on HIV incidence in the opposite gender. The association of population-level annual HIV incidence with annual HSV-2 incidence/prevalence was analysed by calculating crude and adjusted regression coefficients and 95% confidence intervals using linear regression. The effect of HSV-2 on HIV incidence was adjusted for population-level measures of the other factors that were found to be independently associated with HIV incidence at the individual level. In this population, almost all HIV transmission occurs through heterosexual contact (White *et al*. 2007). Therefore, the effect of HSV-2 on HIV acquisition in the population was investigated by examining how HIV incidence in a given gender is related to HSV-2 in the same gender. The effect of HSV-2 on HIV transmission was investigated by examining how HIV incidence in one gender is influenced by HSV-2 in the opposite gender. As HSV-2 is more transmissible than HIV, it was hypothesised that at a population level, changes in HSV-2 incidence are likely to precede changes in HIV incidence. For this reason, analyses examined both the correlation between annual HIV incidence and HSV-2 infection in the same year, and correlations between HIV incidence and HSV-2 infection 1 year and 3 years previously, respectively.

### Ethics approvals

The study obtained approval from the science and ethics review committees of the London School of Hygiene and Tropical Medicine, the Uganda Virus Research Institute and the Uganda National Council for Science and Technology.

## Results

### Study population

Between January 1990 and December 2007, 17 118 participants aged 13–59 years were censused as resident in the study population on at least one annual survey. Of these, 14 016 (81.9%) had one or more HIV test results, of whom 13 021 (92.9%) were HIV negative at their first visit with known HIV status and were eligible for inclusion in HIV incidence analyses. Of these eligible participants, 10 137 (77.9%) had subsequent visits with known HIV status and were included in the incidence analysis. The median follow-up time was 4.2 years (range 0.2–18.0 years) and was similar in men and women (4.6 years *vs*. 4.1 years; *P* = 0.29). Table 1 shows further details of the study population by gender, period and age.

Among the 13 021 HIV-negative participants, those with follow-up HIV data and thus included were more likely to be female, were slightly younger, were more likely to be Muslim, to be never married, and to be HSV-2 seropositive than those without follow-up HIV data.

### Overall HIV incidence

Of the 10 137 participants, 387 seroconverted for HIV during follow-up (incidence rate = 6.02/1000 pyr, 95%CI: 5.45–6.65/1000 pyr). HIV incidence was slightly higher in women than in men (6.48/1000 pyr *vs*. 5.51/1000 pyr; IRR = 1.18, 95% CI: 0.96–1.44). The median age at seroconversion was 28.7 years (range 14.5–59.6 years) and was higher in men than in women (31.1 *vs*. 26.0 years; *P* < 0.001). Among HIV seroconverters, the median time to seroconversion was 5.6 years (range 0.4–17.6 years) and was slightly longer in men than in women (5.6 *vs*. 4.4 years; *P* = 0.04). The median time between last negative and first positive test was 1.3 years (range 0.2–18.0 years).

### Trends in HIV incidence

**Figure 1:**
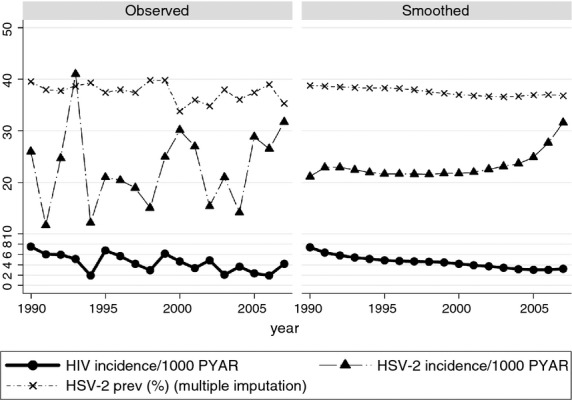
HIV incidence HSV-2 incidence and prevalence in men.

**Figure 2:**
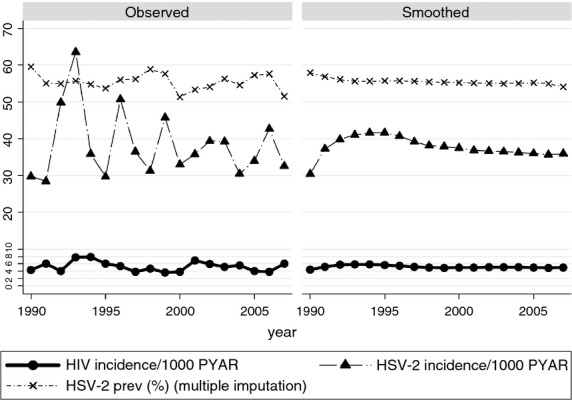
HIV incidence HSV-2 incidence and prevalence in women.

The estimated HIV incidence among men declined fairly steadily from 8.72/1000 pyr in 1990 to 4.85/1000 pyr in 2007 (*P*-trend <0.001; Figure 1), and this was reflected in all age groups. There was less evidence of a decline in incidence among women, that is, 4.86/1000 pyr in 1990 to 6.74/1000 pyr in 2007 (*P*-trend = 0.18; Figure 2).

### Association between HIV incidence and HSV-2 prevalence/incidence

HIV incidence fell over the years (as compared to 1990–1994, IRR = 1.16 [95% CI: 0.79–1.70] in 1995–1999, IRR = 0.78 [95% CI: 0.52–1.19] in 2000–2004, IRR = 0.50 [95% CI: 0.27–0.92] in 2005–2007, *P* = 0.02), increased with age (as compared to aged 13–19 years, IRR = 6.07 [95% CI: 2.93–12.55] among those aged 20–24, IRR = 9.79 [95% CI: 4.62–20.76] among those aged 25–29, IRR = 4.59 [95% CI: 2.14–9.84] among those aged 30–59, *P* < 0.001), and with being an internal migrant within the study villages (IRR = 1.57 [95% CI: 1.09–2.26], *P* = 0.03, i.e. as compared to being a usual resident). Being Muslim was protective (IRR = 0.37 [95% CI: 0.22–0.63], *P* < 0.001), as was being presently or previously married, that is, as compared to having never been married (IRR = 1.05 [95% CI: 0.65–1.70] among those married for 1 + year, IRR = 2.42 [95% CI: 1.44–4.08] among those previously married) and having HSV-2 infection (IRR = 4.03 [95% CI: 1.71–9.50] for incident HSV-2, IRR = 3.01 [95% CI: 1.83–4.94] for prevalent HSV-2).

In women, HIV incidence fell with increasing age (as compared to aged 13–19 years, IRR = 1.48 [95% CI: 0.99–2.19] among those aged 20–24, IRR = 1.17 [95% CI: 0.73–1.86] among those aged 25–29 and IRR = 0.52 [95% CI: 0.33–0.82] among those aged 30–59, *P* < 0.001) was associated with being an internal migrant (IRR = 1.97 [95% CI: 1.42–2.73], *P* < 0.001), belonging to tribe other than Muganda (IRR = 1.41 [95% CI: 1.03–1.91], *P* = 0.03), not being Muslim (IRR = 0.59 [95% CI: 0.41–0.86], *P* = 0.004), being currently or previously married (IRR = 1.79 [95% CI: 0.88–3.64] for currently married and IRR = 0.86 [95% CI: 0.56–1.34] for previously married, *P* = 0.01) and being HVS-2 positive (IRR = 7.25[95% CI: 3.77–13.95] for incident HSV-2 and IRR = 3.71 [95% CI: 2.23–6.18] for prevalent HSV-2, *P* < 0.001).

### Fraction of incident HIV attributable to HSV-2

Population attributable fractions by calendar period and gender ranged between 2–16% for incident HSV-2 infection and 36–65% for prevalent HSV-2 infection (Table 2). PAFs were generally higher for younger age groups (<28 years) than older age groups, that is, 28 year and above (55–79% *vs*. 34–70%, respectively) but were similar in men and women. In both men and women, PAFs were generally lower for incident HSV-2 than for prevalent HSV-2 infection.

**Table 1:** Prevalence and incidence of HSV-2 and incidence of HIV in men and women

	HSV-2 Prevalence (Number at risk)	HSV-2 Incidence cases/pyr	HIV incidence cases/pyr
Men			
Period			
1990–1994	41.0 (1083)	78/3404	45/6984
1995–1999	38.9 (1067)	94/4662	62/8851
2000–2004	28.5 (1485)	109/4933	44/9550
2005–2007	30.8 (1539)	60/2107	16/4945
Age			
13–19	10.2 (2205)	120/7793	10/11 261
20–24	20.7 (641)	80/2738	36/4554
25–29	42.7 (480)	57/1649	46/3415
30–59	64.6 (1848)	84/2926	75/11 099
Women			
Period			
1990–1994	62.4 (1118)	104/2420	61/7656
1995–1999	55.3 (1303)	132/3385	57/9573
2000–2004	48.0 (1799)	146/4103	71/10 827
2005–2007	50.5 (2060)	70/1878	31/5900
Age			
13–19	15.3 (2197)	193/6601	58/10 104
20–24	50.9 (827)	125/2176	59/4962
25–29	66.5 (713)	63/1114	39/4108
30–59	82.2 (2543)	71/1894	64/14 781

### Association between annual HIV incidence and annual HSV-2 prevalence/incidence

There was little evidence of an effect of either annual HSV-2 incidence or prevalence in the previous year or 3 years previously, on annual HIV incidence (Table 3), although there was some evidence that HIV incidence in men was associated with HSV-2 incidence in women 3 years previously; and that HIV incidence in women was associated with HSV-2 incidence in both men and women in the previous year (Table 3). However, after adjusting for other population-level factors, that is, age, being an internal migrant, not being Muslim, being presently or previously married, and being HSV-2 infected, as well as time period (men only) and belonging to tribe other than Muganda (women only), HIV incidence in men was only associated with overall HSV-2 incidence (regression coefficient 0.1, 95%CI: 0.03–0.2, *P* = 0.04), and HIV incidence in women was only associated with HSV-2 incidence in men in the previous year (regression coefficient 0.1, 95%CI: 0.02–0.2, *P* = 0.04; Table 3).

**Table 2:** Population attributable fraction of HIV incidence attributable to HSV-2 infection, by age, sex and period*

	PAFs [95% CI]					
	1990–1994	1995–1999	2000–2004	2005–2007	Overall
Men and women					
Aged below 28 years					
Incident	3	16	9	15	10
Prevalent	51	60	68	52	62
Overall	55 [2, 79]	79 [54, 90]	79 [59, 89]	75 [12, 93]	75 [63, 83]
Aged 28 years and above					
Incident	0	21	−1	20	6
Prevalent	52	−7	69	20	30
Overall	52 [−54, 85]	−4 [NE]	70 [0, 91]	35 [−105, 79]	34 [2, 56]
Men and women, overall					
Incident	2	15	10	16	12
Prevalent	47	39	65	36	51
Overall	50 [10, 72]	55 [30, 72]	75 [56, 85]	57 [1, 81]	64 [51, 73]
Men only					
Aged below 30 years					
Incident	13	9	−1	26	6
Prevalent	18	67	71	41	61
Overall	39 [−111, 82]	76 [32, 92]	67 [14, 87]	73 [≤10, 99]	68 [41, 82]
Aged 30 years and above					
Incident	−1	30	−1†	−2	9
Prevalent	63	6	52†	33	35
Overall	63 [−138, 94]	18 [−52, 56]	39 [−65, 77]	33 [−313, 89]	41 [−2, 66]
Men, overall					
Incident	10	18	−1	10	7
Prevalent	39	44	68	43	53
Overall	48 [−35, 80]	62 [26, 80]	64 [24, 83]	56 [≤10, 100]	61 [40, 74]
Women only					
Aged below 25 years					
Incident	−1	12	11	18	10
Prevalent	57	57	71	38	64
Overall	54 [−18, 82]	72 [25, 89]	86 [60, 95]	70 [−31, 93]	76 [59, 86]
Aged 25 years and above					
Incident	−1	25	15	18	17
Prevalent	42	−11	62	21	30
Overall	42 [−146, 86]	−9 [NE]	74 [−56, 96]	32 [−186, 84]	40 [−9, 67]
Women, Overall					
Incident	−2	15	15	19	14
Prevalent	46	41	66	35	53
Overall	44 [−14, 73]	57 [18, 77]	82 [58, 92]	59 [−11, 85]	67 [52, 78]

NE, Note estimated because computationally minute.

* Adjusted by residence status, religion, marital status and age (if not categorised by age).

† Not adjusted by marital status as all HIV seroconverters were married.

**Table 3:** Association between annual HIV incidence and HSV-2 incidence and prevalence

	Previous year Coefficient – crude [95% CI]†	Previous year Coefficient – adjusted [95% CI]†	Three years previously Coefficient – crude [95% CI] †	Three years previously Coefficient – adjusted [95% CI] †
Men				
HSV-2 incidence in men	−0.1 [−0.1, 0.04]	−0.1 [−0.2, 0.03 ]	0.1 [−0.04, 0.2]	0.1 [−0.004, 0.3]
HSV-2 incidence in women	−0.1 [−0.1, 0.02]	−0.04 [−0.1, 0.05]	0.1 [0.01, 0.1], *P* = 0.05	0.1 [−0.01, 0.2]
HSV-2 incidence overall	−0.1 [−0.1, 0.02]	−0.1 [−0.2, 0.04]	0.1 [−0.02, 0.2]	0.1 [0.03, 0.2], *P* = 0.04
HSV-2 prevalence in men	0.5 [0.1, 0.9]	0.4 [−0.2, 0.9]	0.4 [−0.1, 0.8]	0.1 [−0.5, 0.7]
HSV-2 prevalence in women	0.1 [−0.2, 0.5]	0.1 [−0.3, 0.6]	0.3 [−0.1, 0.6]	0.3 [−0.2, 0.8]
HSV-2 prevalence overall	−0.1 [−0.3, 0.1]	−0.02 [−0.4, 0.4]	0.0 [−0.2, 0.2]	0.1 [−0.4, 0.6]
Women				
HSV-2 incidence in men	0.1 [0.01, 0.1], *P* = 0.06	0.1 [0.02, 0.2], *P* = 0.04	0.1 [0, 0.2]	−0.1 [−0.2, 0.1]
HSV-2 incidence in women	0.1 [0.02, 0.1], *P* = 0.01	0.1 [−0.01, 0.1]	0. 0 [−0.1, 0.1]	−0.04 [−0.1, 0.03]
HSV-2 incidence overall	0.1 [0.02, 0.1], *P* = 0.02	0.1 [−0.002, 0.2]	0. 0 [−0.1, 0.1]	−0.02 [−0.1, 0.1]
HSV-2 prevalence in men	−0.1 [−0.5, 0.3]	−0.4 [−0.9, 0.1]	0.2 [−0.2, 0.6]	0.1 [−0.4, 0.5]
HSV-2 prevalence in women	−0.2 [−0.5, 0.1]	−0.2 [−0.6, 0.1]	0.2 [−0.1, 0.6]	0.2 [−0.2, 0.6]
HSV-2 prevalence overall	0.001 [−0.2, 0.2]	0.1 [−0.3, 0.4]	−0.2 [−0.3, −0.02], *P* = 0.04	−0.1 [−0.4, 0.3]

† *P*-value >0.05 if not indicated.

Adjusted by median age, per cent of population that were internal migrants, per cent that were not Muslim, per cent that had ever been married and per cent that were HSV-2 infected.

Each coefficient represents the direction and magnitude of effect of HSV-2 prevalence/incidence on HIV incidence. For example, an increase of 1/1000 pyr in HSV-2 incidence in men resulted in a 0.1/1000 pyr increase in the HIV incidence in women.

## Discussion

Ours is the first study in sub-Saharan Africa to have long-term, annual data on HSV-2 and HIV infections, enabling analysis of effects of HSV-2 on HIV incidence in relation to time, a subject that is otherwise often studied using mathematical models because of lack of actual observed data.

### Individual-level association between HIV incidence and HSV-2 prevalence/incidence

In keeping with previous observational epidemiological studies, HIV seroconversion was independently associated with HSV-2 infection, and risk was higher for incident than for prevalent HSV-2 (del Mar Pujades Rodríguez *et al*. 2002; Reynolds *et al*. 2003; Freeman *et al*. 2006; Glynn *et al*. 2009).

### Fraction of incident HIV attributable to HSV-2

Assuming a causal relationship between HSV-2 infection and HIV incidence, the estimated fraction of incident HIV attributable to HSV-2 infection in this population where HIV prevalence ranged between 6.2% and 8.5% is high (Shafer *et al*. 2008). Overall, PAFs ranged between 50% and 75% and were higher for prevalent than for incident HSV-2, were higher for younger than for older people, but were similar in women and men. PAFs were persistently high and did not show any pattern of decline or increase with time. PAFs are a function of the proportion of the population with the risk factor being investigated. This explains the higher PAFs for prevalent HSV-2 as the proportion of prevalent HSV-2 in the population is higher than that for incident HSV-2. The frequency of HSV-2 reactivations is known to decrease over time, and this coupled with the higher incidence in the young people may explain the lower PAFS in older people (Benedetti *et al*. 1999).

Population attributable fractions in other population-based studies vary, from 15% to 21% for genital ulcer disease in Rakai district, Uganda (Gray *et al*. 1999), and 59–65% for HSV-2 infection in Mwanza region, Tanzania (Todd *et al*. 2006). The low PAFs in the Rakai study may be partly due to misclassification of HSV-2 positives without GUD as HSV-2 negative. The high PAFs in Mwanza are likely to reflect more sensitive testing for HSV-2, although in contrast the PAFs from Mwanza were higher in men than in women. Studies using mathematical models using data from Cotonou, Benin and Kisumu, Kenya have estimated that the proportion of incident HIV attributable to HSV-2 increases with time as the HIV epidemic unfolds and estimated PAFs ranging between 25% and 50% in mature epidemics (Freeman *et al*. 2007; Abu-Raddad *et al*. 2008).

Despite the likely importance of HSV-2 as a driver of the HIV epidemic, at present, there is no effective or feasible HSV-2 intervention against HIV acquisition. However, there is some evidence from mathematical modelling studies that an effective population-wide intervention against HSV-2 may reduce HIV incidence rates (White *et al*. 2008; Freeman *et al*. 2009). There is need for research on effective HSV-2 interventions that can be used for HIV prevention. These might include higher-dose acyclovir or valacyclovir or a vaccine. In addition, further studies into the biological mechanisms by which HSV-2 may increase HIV risk are necessary.

### Relative trends of HIV incidence and HSV-2 prevalence/incidence

From the mid-1990s, HSV-2 incidence increased in both sexes, while HIV incidence continued to decrease in men and remained roughly constant in women. This divergence may reflect differences in transmissibility and risk factors for these two STIs. HSV-2 is a good biomarker of risky sexual behaviour, particularly among young adults (Cowan *et al*. 1994; Obasi *et al*. 1999). The lack of decrease in HSV-2 incidence therefore suggests persistent risky sexual behaviour. Increases in HSV-2 incidence in the face of declining HIV incidence may have been because HSV-2 is more easily transmissible and had higher background prevalence than HIV, therefore a modest increase in risk behaviour may have resulted into reversal of a declining trend in HSV-2 incidence without substantially changing trend of HIV incidence. In addition, the prevalence of curable STIs may have decreased with increased availability of syndromic STI treatment in the population (Morgan *et al*. 2001). This may have reduced HIV incidence without affecting HSV-2 incidence.

At population level, an effective intervention for both HIV and HSV-2 is likely to achieve reductions in HIV incidence ahead of reductions in HSV-2 because of higher transmissibility and higher background prevalence of HSV-2. For the same reason, increase in risky sexual behaviour is likely to manifest as increase in HSV-2 incidence before increase in HIV incidence, and HIV incidence may not increase if the risky behaviour is short-lived. Furthermore, even for an intervention with similar impact of both infections, a change in HSV-2 incidence is likely to be detected earlier because HSV-2 has a higher background incidence rate as compared to HIV.

Only one study from Africa has compared trends in prevalence of HIV and HSV-2. The study tested stored blood samples from population-based controls in TB case–control studies from 1988 to 2005 in Karonga district in northern Malawi. In the respective study periods, HIV prevalence increased from 4.0% to 12.3% in men and 4.3% to 16.3% in women. In contrast, HSV-2 prevalence remained roughly constant in men at around 30% and in women at around 40–50% (Glynn *et al*. 2008).

### Population-level association between HIV incidence and HSV-2 prevalence/incidence

Overall, there was little evidence for an association between annual HIV incidence and annual HSV-2 incidence/prevalence, but data were sparse and 95% CIs wide. Adjusted analyses found modest associations, that is, HIV incidence in men with overall HSV-2 incidence and HIV incidence in women with HSV-2 incidence in men in the previous year. Given the strong association between HIV incidence and HSV-2 at the individual level, this result is unexpected. It is possible that there was insufficient variation in annual HIV incidence or HSV-2 prevalence and incidence to demonstrate an association. It appears that modest changes in prevalence or incidence of HSV-2 are likely to have little effect on annual HIV incidence in the short term. It may take several years before any changes in HSV-2 epidemiology affect HIV incidence as shown by the modelling studies (White *et al*. 2008; Freeman *et al*. 2009). Also, in this ecologic study design, population-level data may have failed to adequately represent individual characteristics thereby failing to demonstrate the association seen at individual-level analyses (Haneuse & Bartell 2011).

### Limitations

Selection bias arising both from lack of follow-up of HIV results and from loss to follow-up due to out-migration or non-participation cannot be completely excluded. Even in this long-standing study, median follow-up time for participants followed for seroconversion was only about 4 years. About 22% of participants eligible for follow-up for HIV seroconversion did not have results after their first known HIV-negative result. As compared to the participants included in the incidence analysis, the participants excluded because of lack of follow-up HIV results, were more likely to be female, HSV-2 positive and previously married. As these characteristics are associated with a higher HIV incidence rate, the estimated incidence may be an underestimate. However, these participants (excluded because of lack of follow-up HIV results) were also more likely to be younger, Muslims and never married – all of whom tend to have lower HIV incidence. Therefore, the overall effect of the differences in distribution in the characteristics of participants followed for HIV seroconversion and those excluded because of lack of follow-up HIV results may have been minimal.

In summary, over the study period, declines in HIV incidence were seen in men but not in women. There is need for renewed vigilance for prevention efforts. Known prevention methods such as condom promotion, promotion of abstinence and faithfulness and STI treatment should be renewed, as well as adoption of newer methods such as voluntary medical male circumcision and early HAART in serodiscordant couples. Further, assuming a causal relationship, 50–75% of new HIV infections in this study population are attributable to prevalent HSV-2. An intervention that effectively prevents acquisition of HSV-2 or suppresses HSV-2 reactivation may help prevent many new HIV infections in populations similar to this. Effort to develop an effective HSV-2 intervention should be strongly supported despite lack of efficacy of previous RCTs.
